# Sequence and expression analysis of HSP70 family genes in *Artemia franciscana*

**DOI:** 10.1038/s41598-019-44884-y

**Published:** 2019-06-10

**Authors:** Wisarut Junprung, Parisa Norouzitallab, Stephanie De Vos, Anchalee Tassanakajon, Dung Nguyen Viet, Gilbert Van Stappen, Peter Bossier

**Affiliations:** 1 Laboratory of Aquaculture & Artemia Reference Center, Department of Animal Sciences and Aquatic Ecology, Faculty of Bioscience Engineering, Ghent University, Coupure Links 653, 9000 Ghent, Belgium; 2 Center of Excellence for Molecular Biology and Genomics of Shrimp, Department of Biochemistry, Faculty of Science, Chulalongkorn University, Bangkok, 10330 Thailand; 3 Laboratory for Immunology and Animal Biotechnology, Department of Animal Sciences and Aquatic Ecology, Faculty of Bioscience Engineering, Ghent University, Coupure 653, Ghent, 9000 Belgium; 4 Present Address: Research Institute for Aquaculture No. 2, Ho Chi Minh City, 71007 Vietnam

**Keywords:** Biochemistry, DNA, Molecular biology

## Abstract

Thus far, only one gene from the heat shock protein 70 (*HSP70*) family has been identified in *Artemia franciscana*. Here, we used the draft *Artemia* transcriptome database to search for other genes in the HSP70 family. Four novel HSP70 genes were identified and designated *heat shock cognate 70* (*HSC70*), *heat shock 70* *kDa cognate 5* (*HSC70-5*), *Immunoglobulin heavy-chain binding protein (BIP)*, and *hypoxia up-regulated protein 1* (*HYOU1*). For each of these genes, we obtained nucleotide and deduced amino acid sequences, and reconstructed a phylogenetic tree. Expression analysis revealed that in the juvenile state, the transcription of *HSP70* and *HSC70* was significantly (*P* < 0.05) higher in a population of *A*. *franciscana* selectively bred for increased induced thermotolerance (TF12) relative to a control population (CF12). Following non-lethal heat shock treatment at the nauplius stage, transcription of *HSP70*, *HSC70*, and *HSC70-5* were significantly (*P* < 0.05) up-regulated in TF12. In contrast, transcription of the other HSP70 family members in *A*. *franciscana* (*BIP*, *HYOU1*, and *HSPA4*) showed no significant (*P* > 0.05) induction. Gene expression analysis demonstrated that not all members of the HSP70 family are involved in the response to heat stress and selection and that especially altered expression of *HSC70* plays a role in a population selected for increased thermotolerance.

## Introduction

Heat shock proteins (HSPs) are a family of highly conserved proteins which expression responds to environmental stressors, such as high temperature, ultraviolet light, inflammation, infection and cellular toxins. HSPs are usually named according to their molecular weights; small heat shock proteins (sHSPs) have a molecular mass of 18–40 kDa while major HSPs have a variety of molecular masses, including HSP60, HSP70, HSP90 and HSP100^1^. HSPs play essential roles in organisms, particularly in terms of protection against a variety of stressors. Heat stress can disturb cellular homeostasis in all organisms, potentially resulting in death^2^. However, to mitigate such stress, metabolic profiles often undergo changes so that metabolic homeostasis can be re-established rapidly and effectively; for example, in order to repair cellular damage. Previous research has shown that HSP proteins play a key role in these metabolic changes^3^.

The HSP70 family is a group of stress response proteins that has been studied extensively in prokaryotic and eukaryotic organisms^3^. The number of HSP70 family members expressed is species-dependent. The human HSP70 family features 13 genes that differ from each other in terms of expression level, subcellular location and amino acid constitution^4^. However, in general, the HSP70 family can be divided into four groups. The first group is HSP70, which is either not or moderately expressed, under normal conditions. However, the expression of HSP70 can be rapidly induced when cells are exposed to heat, cold or other stressors^5,6^. The second group is HSC70, which is expressed constitutively in most human tissues. In humans, HSC70 exhibits 86% identity to HSP70^7^. According to previous reports, the expression of HSC70 can also be induced by heat stress^8^, but again that can be species-dependent. The third group is glucose-regulated protein 78 (GRP78), which is also known as immunoglobulin heavy-chain binding protein (BIP) or heat shock 70 kDa cognate 3. GRP78 is an important endoplasmic reticulum (ER) chaperone that is localised to the ER and plays essential roles in the unfolded protein response (UPR), oxidative stress, ER calcium binding and the activation of transmembrane ER stress sensors. GRP78 can be induced by stressors such as salinity, pH and excessive exposure to metal^7,9^. The fourth group is glucose-regulated protein 76 (GRP75) which is predominantly expressed in the mitochondria and is not induced in response to stress^7^.

Thus far, only one gene of the HSP70 family, namely HSP70 has been described in *Artemia franciscana* Kellogg, 1906 (accession number: AF427596). Acknowledging that in all eukaryotes this family has multiple members, it is of particular interest to investigate this family of genes in stress tolerant organism as *Artemia*. In this study, novel members of the HSP70 family were identified from a draft *Artemia* genome database (fully annotated but unpublished yet) using bioinformatic tools. Orthology prediction methods analysed the complete open reading frame (ORF) for each gene identified. Gene sequences were then subjected to phylogenetic analysis. Next, we investigated the expression of the identified HSP70 members in two populations of *Artemia*: a control population (CF12) that experienced 12 generations of isothermal laboratory culture conditions and a selected population (TF12) featuring the survivors of over 12 generations of an induced thermotolerance treatment. In addition, the expression of the newly identified HSP70 members was also examined following non-lethal heat shock (NLHS) in both CF12 and TF12 populations. Our results showed that members of the HSP70 family respond differentially at the transcriptional level to “thermal” selection or to NLHS, or to both.

## Results

### Sequence identification and phylogenetic analysis of genes from the HSP70 family

To identify HSP70 family members in *A*. *franciscana*, we used reference HSP70 genes from closely related species (Table S1) to search an *Artemia* transcriptomic database. Partial cDNA sequences of HSP70 genes were then confirmed by PCR amplification using juvenile *Artemia* (SFB, ARC1768) and DNA sequencing. Sequence analysis identified four members of the HSP70 family with a complete ORF containing putative conserved HSP70 domains: *heat shock cognate 70* (*HSC70*); *heat shock 70* *kDa cognate 5* (*HSC70-5*); *heat shock 70* *kDa cognate 3* (*HSC70-3*)/*BIP*/*Grp78* and *hypoxia up-regulated protein 1* (*HYOU1*). A partial sequence for *HSPA4* was also identified and reported below.

### Heat shock cognate 70 (HSC70)

**Figure 1:**
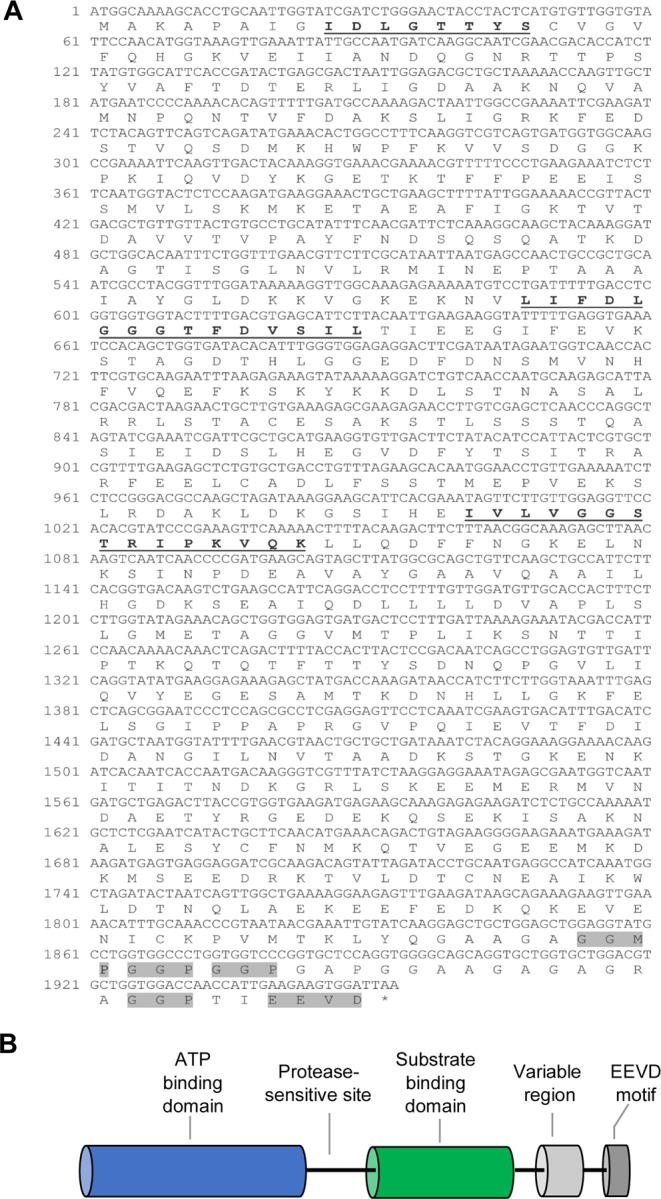
Schematic diagram and domain analysis representing the complete open reading frame (ORF) of *A*. *franciscana* HSC70. (**A**) Nucleotide and deduced amino acid sequences and (**B**) cartoon showing a linear representation of HSC70. The three HSP70 protein family signatures are labeled with bold letters and are underlined. The C-terminal region containing the tetrapeptide GG[X]P and an EEVD-motif is labeled with a grey background.

**Figure 2:**
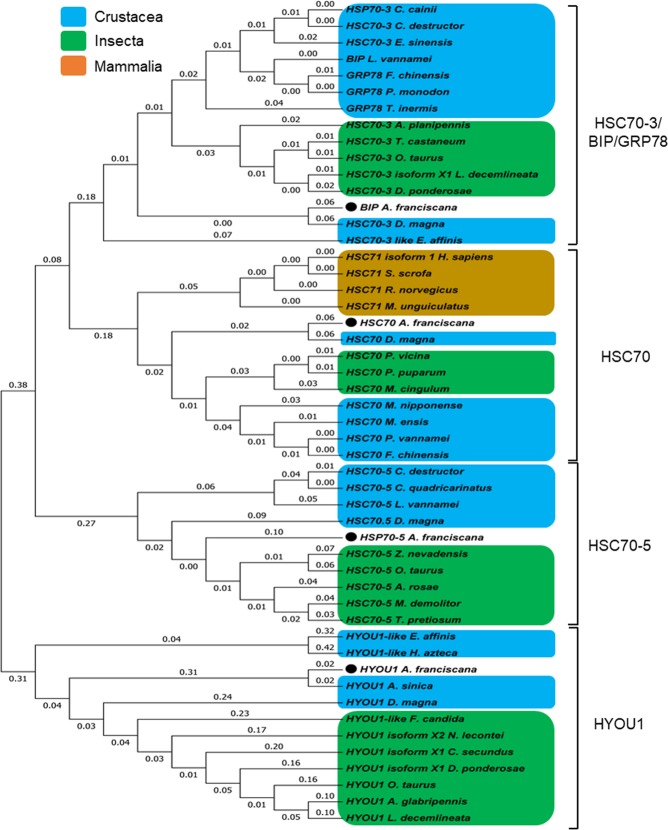
Phylogenetic tree of HSP70 family members in *A*. *franciscana*. The neighbour-joining phylogenetic tree of HSP70 genes was created using their amino acid sequences. Phylogenetic tree analysis of the partial or complete open reading frame (ORF) amino acid sequences of five novel HSP70 genes were aligned with ClustalW and used to reconstruct a phylogenetic tree using the neighbor-joining method and MEGA 7.0 software. The GeneBank numbers were listed as follows: **HSC70-3/BIP/GRP78** (*Cherax cainii*: AKB96213.1; *Cherax destructor*: AKB96212.1; *Eriocheir sinensis*: AHA61465.1; *Litopenaeus vannamei*: AFQ62791.1.); *Fenneropenaeus chinensis*: ABM92447.1; *Penaeus monodon*: ARW29625.1; *Thysanoessa inermis*: ARN17957.1; *Agrilus planipennis*: XP_018330023.1; *Tribolium castaneum*: XP_008200986.2; *Onthophagus Taurus*: XP_022900037.1 *Leptinotarsa decemlineata*: XP_023015278.1; *Dendroctonus ponderosae*: XP_019762356.1; *Artemia franciscana*: MH992635; *Daphnia magna*: A0A0N8CDY8; *Eurytemora affinis*: XP_023346701.1), **HSC70** (*Homo sapiens*: NP_006588.1; *Sus scrofa*: NP_001230836.1; *Rattus norvegicus*: NP_077327.1; *Meriones unguiculatus*: XP_021498722.1; *Artemia franciscana*: MH992633; *Daphnia magna*: A0A0P5G8T5; *Polyrhachis vicina*: AGF33487.1; *Pteromalus puparum*: ACA53150.1; *Macrocentrus cingulum*: ACD84943.1; *Macrobrachium nipponense*: ABG45886.1; *Metapenaeus ensis*: ABF20530.1; *Litopenaeus vannamei*: ABP01681.1; *Fenneropenaeus chinensis*: AAW71958.1), **HSC70-5** (*Cherax destructor*: AKB96210.1; *Cherax quadricarinatus*: AKB96209.1; *Litopenaeus vannamei*: ANJ04741.1; *Daphnia magna*: KZS16423.1; *Artemia franciscana*: MH992632; *Zootermopsis nevadensis*: XP_021939160.1; *Onthophagus taurus*: XP_022919865.1; *Athalia rosae*: XP_012264348.1; *Microplitis demolito*: XP_008560903.1; *Trichogramma pretiosum*: XP_014221706.1), and **HYOU1** (*Hyalella Azteca*: XP_018009810.1; *Artemia franciscana*: 992634; *Artemia sinica*: AKG51639.1; *Daphnia magna*: A0A0P5LA98; *Eurytemora affinis*: XP_023323737.1; *Folsomia candida*: XP_021945992.1; *Neodiprion lecontei*: XP_015511534.1; *Cryptotermes secundus*: XP_023720786.1; *Anoplophora glabripennis*: XP_018567874.1; *Leptinotarsa decemlineata*: XP_023015278.1; *Dendroctonus ponderosae*: XP_019762356.1; *Onthophagus Taurus*: XP_022900037.1).

The complete ORF of *HSC70* is 1953 bp in length (accession number: MH992632) and encodes a putative protein of 650 amino acids (Fig. 1A). This sequence contains a typical ATP-binding domain and a substrate-binding domain of the HSP70 family (Fig. 1B). The deduced amino acid sequence of *HSC70* includes the three signature sequences of genes from the HSP70 family including IDLGTTYS (residues 9–16), LIFDLGGGTFDVSIL (residues 196–210) and IVLVGGSTRIPKVQK (residues 334–348), as predicted by ExPASy software. Moreover, a series of unique repeat GG[X]P motifs, and a cytosolic Hsp70–specific motif, EEVD (residues 647–650), were found at the C-terminus of HSC70 (Fig. 1A). The protein has a molecular mass of 70.89 kDa and theoretical isoelectric point (pI) of 5.38, as predicted by ExPASy tools. A phylogenetic tree was reconstructed based on the deduced amino acid sequence of the complete HSC70 ORFs. The phylogenetic tree featured three main classes (insects, crustaceans, and mammals; Fig. 2) and revealed that HSC70 in *A*. *franciscana* exhibited the highest sequence similarity to HSC70 in *Daphnia magna* (A0A0P5G8T5), with 88% identity.

### Heat shock 70 kDa cognate 5 (HSC70-5)

**Figure 3:**
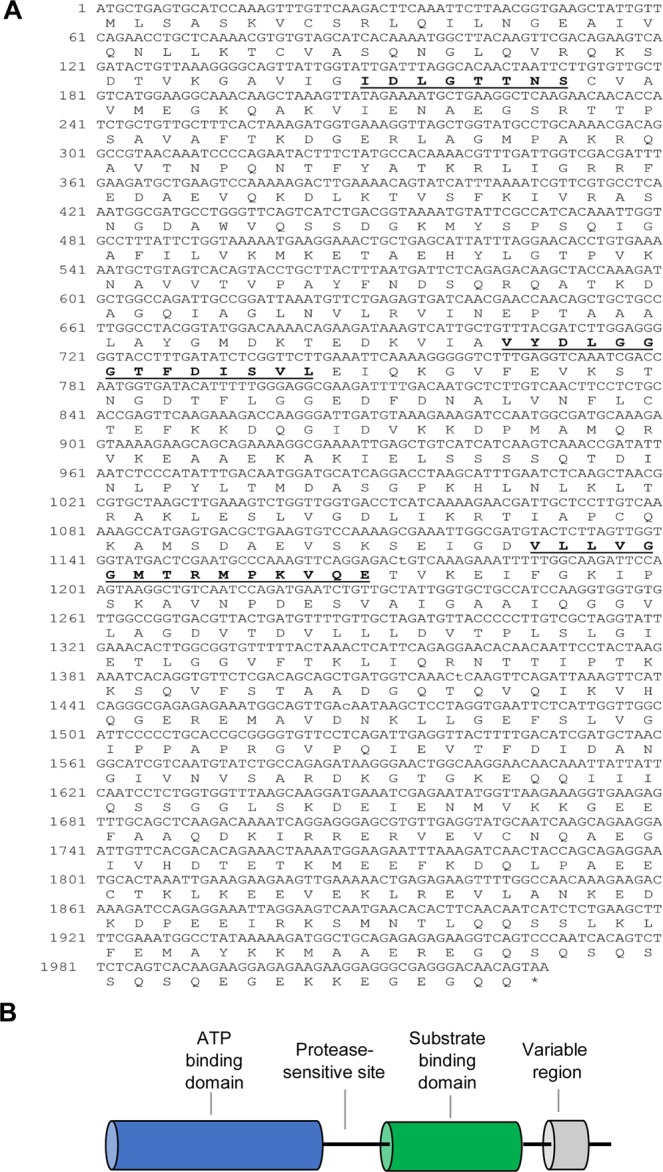
Schematic diagram and domain analysis representing the complete open reading frame (ORF) of *A*. *franciscana* HSC70-5. (**A**) Nucleotide and deduced amino acid sequences and (**B**) cartoon showing a linear representation of HSC70. The three HSP70 protein family signatures are labeled with bold letters and are underlined.

The complete ORF of *HSC70-5* is 2028 bp in length (accession number: MH992633) and translates into a putative protein of 675 amino acids (Fig. 3A). The deduced amino acid sequence contains a typical ATP-binding domain and substrate-binding domain (Fig. 3B). It also includes three signature sequences of HSP70 genes: IDLGTTNS (residues 50–57), VYDLGGGTFDISVL (residues 235–248) and VLLVGGMTRMPKVQE (residues 376–390) (Fig. 3A), as predicted by ExPASy tools. The protein has a molecular mass of 73.30 kDa and a theoretical pI of 5.57, as predicted by ExPASy software. A phylogenetic tree was reconstructed based on the deduced amino acid sequence of the *HSC70–5* complete ORF and showed two different classes: insects and crustaceans (Fig. 2). *Artemia franciscana HSC70-5* was closely related to insect amino acid sequences (Fig. 2), and showed highest sequence similarity to HSC70-5 in *Onthophagus taurus* (XP_022919865.1), with 82% identity.

### Immunoglobulin heavy-chain binding protein (BIP)

**Figure 4:**
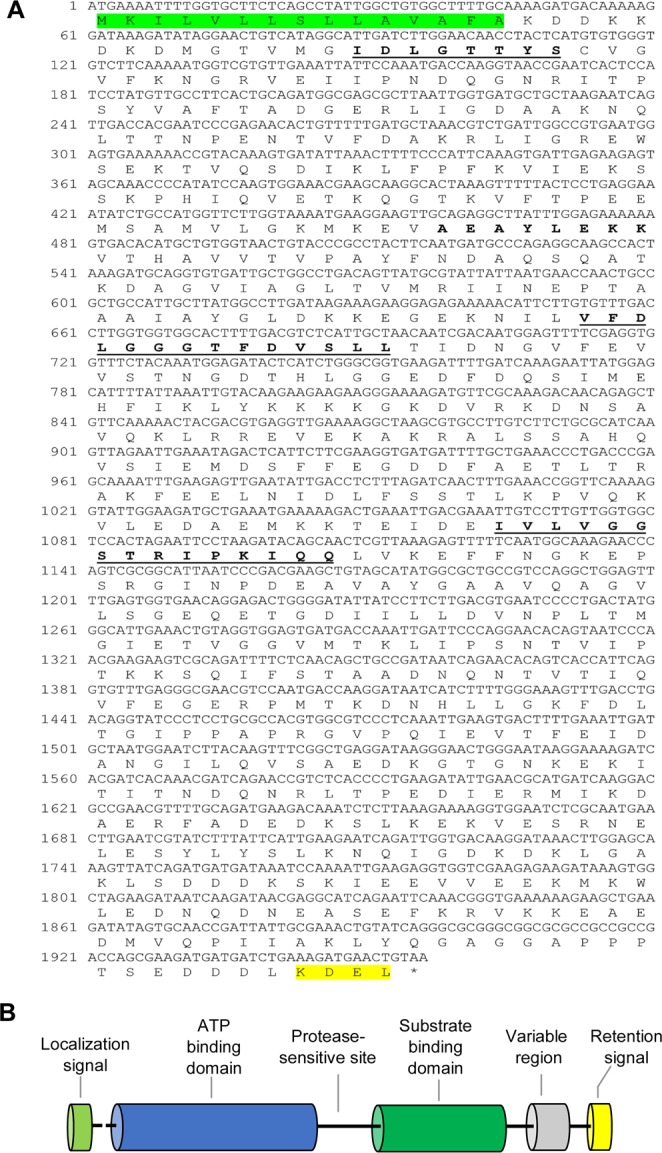
Schematic diagram and domain analysis representing the complete open reading frame (ORF) of *A*. *franciscana* BIP. (**A**) Nucleotide and deduced amino acid sequences and (**B**) cartoon showing a linear representation of HSC70. The signaling peptide is labeled with a green background. The three HSP70 protein family signatures are labeled with bold letters and are underlined. The ATP/GTP-binding site motif A (P-loop) is showed with a bold letter. The C-terminal region containing a KDEL-motif is labeled with a yellow background and represents the endoplasmic reticulum retention sequence.

The complete ORF of *A*. *franciscana BIP* (accession number: MH992635) is 1956 bp in length and encodes a protein of 651 amino acids (Fig. 4A). This protein (Fig. 4B) contains an ATP-binding domain and a substrate-binding domain. The signal peptide for secretion into the ER, 1MKILVLLSLLAVAFA15, is located at the N-terminus. The deduced amino acid sequence of *A*. *franciscana BIP* contains the following conserved signature sequences: IDLGTTYS (residues 30–37), VFDLGGGTFDVSLL (residues 218–231) and IVLVGGSTRIPKIQQ (residues 355–369). Moreover, an ATP/GTP-binding site motif A (P-loop) is located at residues 153–160 (AEAYLEKK) (Fig. 4A). The C-terminus of BIP has a putative ER retention tetrapeptide, KDEL, at residues 648–651, as predicted by ExPASy tools (Fig. 4A,B). The protein has a molecular mass of 72.16 kDa and a theoretical pI of 5.12, as predicted by ExPASy software. A phylogenetic tree of two different classes of insecta and crustaceans is shown in Fig. 2; this revealed that *A*. *franciscana BIP* exhibited the highest sequence similarity to HSC70-3 in *D*. *magna*, with 87% identity.

### Hypoxia up-regulated protein 1 (HYOU1)

**Figure 5:**
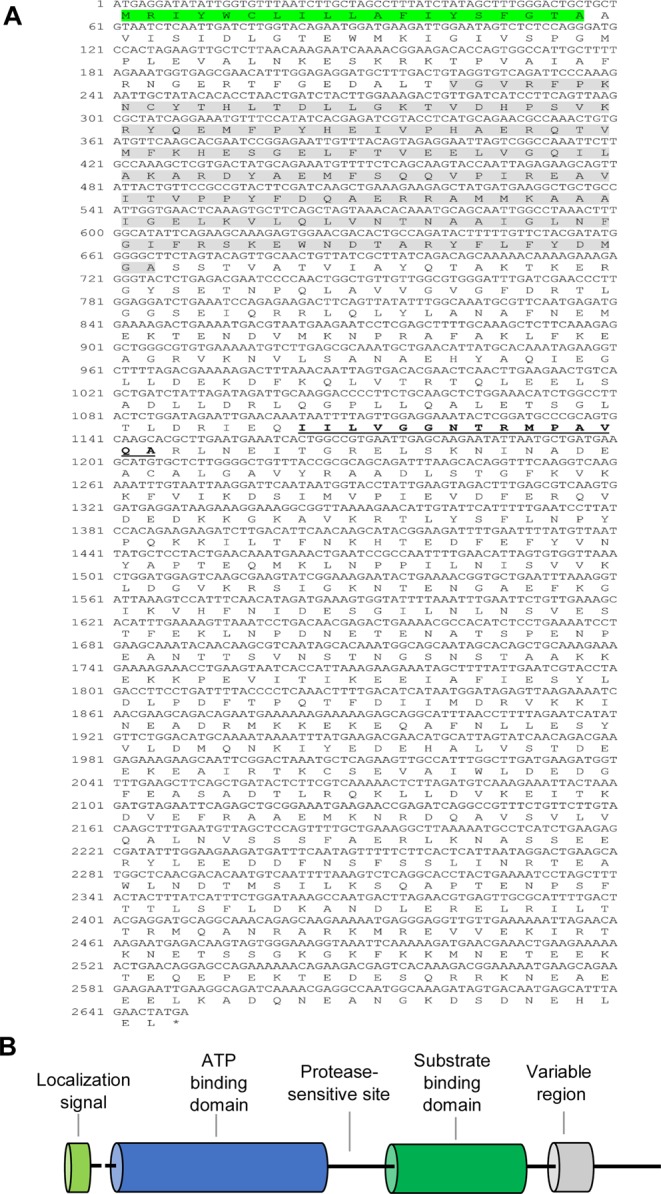
Schematic diagram and domain analysis representing the complete open reading frame (ORF) of *A*. *franciscana* HYOU1. (**A**) Nucleotide and deduced amino acid sequences and (**B**) cartoon showing a linear representation of HSC70. The signaling peptide is labeled with a green background. One HSP70 protein family signature is labeled with a bold letter and is underlined. The HYOU1-like_NBD domain is highlighted with a grey background.

The complete ORF of *HYOU1* (accession number: 992634) is 2649 bp in length and encodes a putative protein with 882 amino acids (Fig. 5A). This protein contains putative conserved domains of the HSP70 superfamily, namely an ATP-binding and a substrate binding domain (Fig. 5B). The signal peptide for secretion into the ER, 1MKILVLLSLLAVAFA15, is located at the N-terminus. One signature motif of HSP70 family genes was found at residue 368–382: IILVGGNTRMPAVQA (Fig. 5A). The protein has a molecular mass of 100 kDa and a theoretical pI of 5.23, as predicted by ExPASy software. A phylogenetic tree was separated into two different classes including insecta and crustaceans; *A*. *franciscana HYOU1* had the highest sequence similarity to *HYOU1* in *Artemia sinica*, with 95% identity (Fig. 2).

### Heat shock 70 kDa protein 4 (HSPA4)

The partial cDNA sequence of HSPA4 is 1269 bp in length (accession number: MK036505). Blast results showed that the partial cDNA of HSPA4 was similar to the HSPA4 isoform, X2, in *Bombus terrestris* with the highest alignment score and 62% identity. Alignment of the amino acid sequence and HSPA4 isoform X2 showed the highest similarity at the N-terminus of HSPA4 from *B*. *terrestris*. The partial HSPA4 protein sequence contains part of an ATP binding domain at the N-terminus. One signature motif of the HSP70 family, VEIVGGSTRIPAVKT, was found at residues 339–353.

### Expression analysis of HSP70 family genes in CF12 and TF12 juveniles

**Figure 6:**
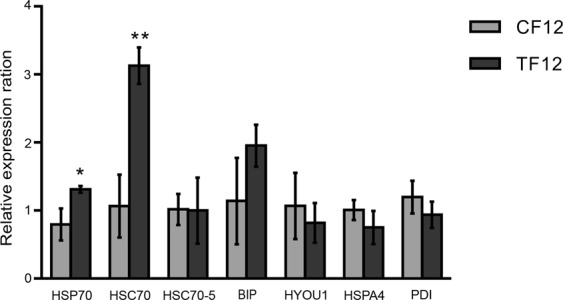
A thermal tolerance selection breeding program induced the expression of HSP70 and HSC70 genes in *A*. *franciscana*. The relative gene expression of HSP70 genes was quantified by qRT-PCR in juvenile *Artemia* from the TF12 population (selective breeding for induced thermotolerance) and compared to that in controls (CF12; non-selective breeding for induced thermotolerance). EF1α was used as an internal control. The error bars represent standard deviation (SD) values from three replicates. Asterisks indicate statistically significant ratios compared with the control (*Indicates *P* < *0*.*05* and **Indicates *P* < *0*.*01*).

The relative gene expression of the newly-identified HSP70 genes was examined in juvenile TF12 and CF12 *Artemia* and analysed by qRT-PCR using specific primers for six HSP70 family genes: *HSP70*, *HSC70*, *HSC70-5*, *BIP*, *HYOU1* and *HSPA4*; an additional primer set was used to amplify *protein disulphide isomerase* (*PDI*). The relative gene expression of the six HSP70 family genes, and one chaperone protein, is shown in Fig. 6. Significantly higher expression of *HSP70* was observed in the TF12 population (*P* < *0*.*05*) as compared to that in the CF12 population. Moreover, a threefold higher expression of *HSC70* was detected in TF12 juveniles compared to CF12 juveniles (*P* < *0*.*01*). On the other hand, *HSC70-5*, *BIP*, *HYOU 1* and *HSPA4* did not show any significant differences in terms of gene expression when compared between CF12 and TF12 juveniles. The relative expression of the control chaperone gene, *PDI*, did not show any significant difference either. In summary, these results suggested that selection for induced thermotolerance in *Artemia* increased the expression of *HSP70* and *HSC70*. Yet more explicitly *HSC70*.

### Expression of HSP70 family genes in CF12 and TF12 nauplii following NLHS

**Figure 7:**
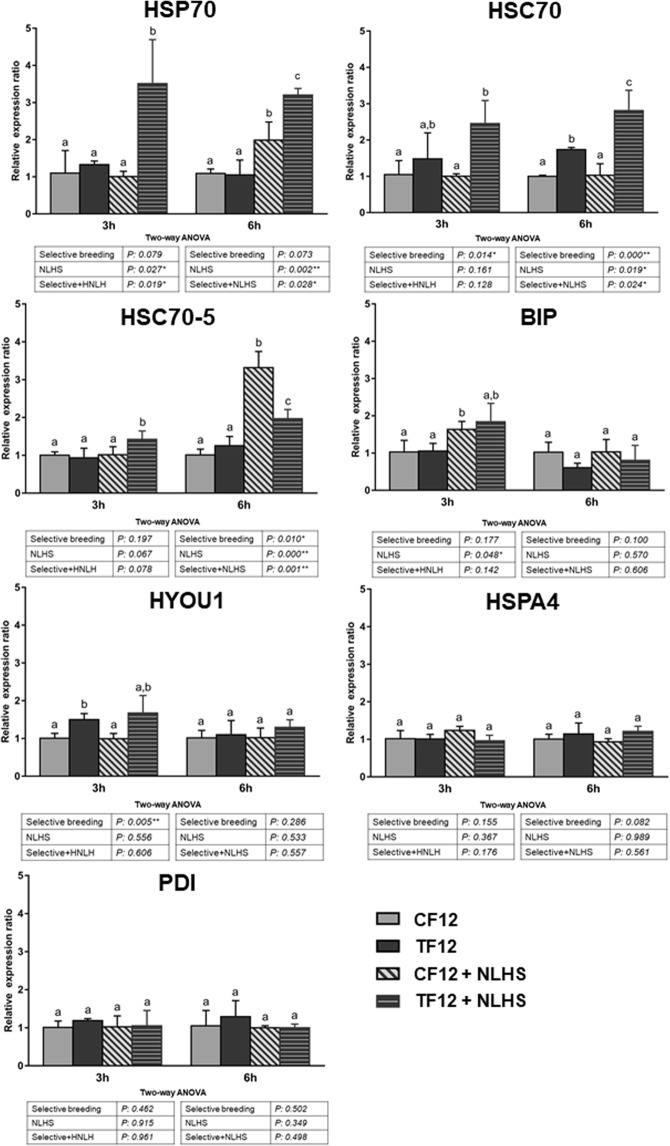
HSP70 gene expression following NLHS treatment in nauplii from CF12 and TF12 populations. Four groups of swimming nauplii from CT12 (non-selective breeding for induced thermotolerance) and TF12 (selective breeding for induced thermotolerance) were treated with NLHS (CF12 + NLHS and TF12 + NLHS) and then animals were collected after 3 and 6 h of recovery to determine the relative gene expression of HSP70 genes (*HSP70*, *HSC70*, *HSC70-5*, *BIP*, *HYOU1* and *HSPA4*), along with the *PDI* gene as a control, by qRT-PCR. EF1α was used as an internal control. The error bars represent standard deviation (SD) values from three replicates. Significant differences are indicated as the by different alphabetic characters and were accepted at *P* < *0*.*05*. Two-way analysis of variance (ANOVA) was performed to investigate the relationship between selective breeding and NLHS factors. Asterisks indicate ratios that were significantly different (*Indicates *P* < *0*.*05* and **Indicates *P* < *0*.*01*).

Next, we investigated whether selected animals responded differently to a non-lethal heat shock (NLHS) when compared to non-selected animals (Fig. 7).

In the control population (CF12), we found that *HSC70-5* and *BIP* (3 h post-NLHS) and *HSP70* expression (6 h post-NLHS) were significantly (*P* < *0*.*05*) up-regulated. However, there was no significant difference in the expression of *HSC70*, *HYOU1*, *HSPA4* or PDI at either of these timepoints following NLHS.

In the TF12 population, NLHS induced significant (*P* < 0.05) changes in the expression of *HSP70*, *HSC70* and *HSC70-5*. At 3 and 6 h post-NLHS, *HSP70* and *HSC70* expression were approximately threefold and twofold higher than non-heat group, respectively. Moreover, *HSC70-5* was slightly up-regulated at 3 and 6 h post-NLHS. Finally, NLHS did not significantly affect the expression of *BIP*, *HYOU1*, *HPA4* and *PDI*.

Two-way ANOVA revealed an interaction between NLHS and selection for induced thermotolerance for *HSP70* (both at 3 and 6 h post-NLHS) and *HSC70* and *HSC70-5* (at 6 h post-NLHS). For *HSC70-5*, the interaction was antagonistic, while in the other two cases, interaction was synergistic. These observations indicate that selection for induced thermotolerance positively interferes with NLHS (at least for *HSP70* and *HSC70*). Remarkably, *HSC70* was not responsive to NLHS in the control population but was sensitive to NLHS in T12 animals. In contrast, for *BIP*, *HYOU1*, *HSPA4* and *PDI*, the effects of NLHS were not dependent on selective breeding for induced thermotolerance (Fig. 7).

## Discussion

Prior to this study, only one HSP70 family gene, *HSP70* (accession number: AF427596), had been identified and reported in *A*. *franciscana*. The *A*. *franciscana HSP70* mRNA sequence, which is 2081 bp in length, was published by Chen and MacRae (2001). In the present study, we identified four novel putative genes from the HSP70 family in *A*. *franciscana*: *HSC70*, *HSC70-5*, *BIP* and *HYOU 1*.

Sequencing analysis showed that HSC70 has high sequence similarity to HSC70 in various organisms. Furthermore, the tetrapeptide GG[X]P and the cytosolic HSC70 specific motif EEVD at the C-terminus were identified in the HSC70 protein sequence. Demand *et al*. (1998) previously reported that cytosolic eukaryotic HSC70 possesses GG[X]P repeats and has an EEVD motif at the carboxy terminus whereas other HSP70 family members lack such structural elements^10^. The function of the tetrapeptide GG[X]P has still to be elucidated. On the other hand, the EEVD motif is known to be involved in the intramolecular regulation of *HSC70*; a previous report found that deletion of the EEVD motif affected ATPase activity and the ability to interact with substrates^10,11^. The creation of a phylogenetic tree revealed that *A*. *franciscana HSC70* was closely related to insect, crustaceans and mammalian *HSC70s*, particularly *Daphnia magna* (with 88% sequence identity). Gene expression analysis showed that there was no significant up-regulation of *HSC70* following NLHS in CF12 animals, suggesting constitutive expression. These results corroborate earlier experiments in humans, which showed that this gene was expressed constitutively in most tissues^12^. Moreover, levels of *HSC70* mRNA in *Tetranychus urticae* (the two-spotted spider mite) were not significantly changed by heat and cold shock treatments^13^. Further experiments in the corn earworm (*Helicoverpa zea*) showed that *HSC70* expression was not induced by heat and cold stress treatments^14^^.^ These results suggest that HSC70 is a cytosolic protein and is constitutively expressed. Moreover, some studies have reported that HSC70 is translocated to the nucleus upon NLHS^3^. In addition, *HSC70-5* was shown to exhibit high sequence similarity with HSC70-5 in the beetle *Ointhophagus taurus* (82% identity) and was induced by heat stress in a manner that was similar to the recently discovered *P*. *vannamei HSC70-5*^15^. In S2 cells, *Lv*HSC70-5 has been shown to localise in mitochondria, where it functions as the regulator of mitochondrial morphology and cellular homeostasis^15,16^. We also identified *BIP* in this study; this was very similar to heat shock protein cognate 3 of *Chelax destructor* (88% identity). *BIP* has been reported to function in the ER lumen and can be induced by environment stressors^5,17^. We also identified *HYOU1*. This protein is grouped into group III of the human HSP70 family and is translated as a 105/110 kDa protein and a 170 kDa protein. *HYOU1* coding for the 170 kDa protein (GRP170) was identified in animals and plants but appears to be absent in other lineages, including fungi^3^. *HYOU1* is an ER-resident chaperone protein, a member of the heat shock protein family, and a member of the ER stress protein families. This gene is localised in the ER and is induced by ER stress^3,18^.

It appeared that some *Artemia* HSP70 genes were closely related to corresponding HSP70 genes in crustaceans. However, some were more closely related to the corresponding HSP70 genes in Hexapoda. This may be because both crustaceans and Hexapoda are classified into the same phylum (Arthropoda). *Artemia franciscana* is classified into the Arthropoda (Phylum), Crustacea (Subphylum) and Branchiopoda (Class). Phylogenetic clustering showed that HSC70, BIP and HYOU1 are closely related to the Malacostraca class of Crustacea; this was consistent with previous results obtained by Regier *et al*. (2010). In a previous study, the alignment of 45 kB DNA sequences from 62 single-copy nuclear protein-coding genes from 75 arthropod species generated a family tree of arthropods in which the Branchiopoda cluster together with Multicrustacea (Copepod, Malacostraca and Thecostraca) in a clade named Vericrustacea (Copepod, Malacostraca, Thecostraca and Branchiopoda)^19^. This suggested that *Artemia* (Branchiopoda) is classified into a similar clade with Malacostraca (shrimp, crab, etc.). On the other hand, *Artemia* HSC70-5 is closely related to insect HSC70-5 (Hexapoda). This means that *Artemia* (Branchiopoda) might also be closely associated with insects, especially the Hexapoda subphylum. This observation is supported by several recent studies^20–25^. Previous research described by Oakley *et al*. (2013) and Eyun (2017) is based on the use of new transcriptomes and a new morphological matrix (including fossils), but also includes existing data relating to expressed sequence tags, the mitochondrial genome, the nuclear genome, ribosomal DNA data and 24 nuclear protein-coding genes; by combining such data, these authors aimed to generate new insights into Pancrustacean phylogeny. Their results revealed that Branchiopoda belong to the clade Allotriocaride, as is the case of Hexapoda, thus explaining why some crustacean genes are closely related to insect genes Hexapoda. However, according to several phylogenetic analyses involving Arthropods^19–25^, we found that the differential classification results for Branchiopoda (*Artemia*), and other classes within the Arthropoda phylum, were dependent upon clusters of input data. For example, phylogenetic results arising from HSC70-5 (mitochondrial HSP) indicated a close relationship with insects, which is consistent with Oakley’s previous results, which were obtained from analysis involving the mitochondrial genome^23^.

A number of previous studies have indicated that a non-lethal heat shock (NLHS) (but also other stressors) can induce the expression and production of HSP70, such as in *Artemia*, *P*. *vannamei*, *P*. *monodon* and *P*. *viridis* (green mussels)^2,26–28^. However, only the expression of one gene, HSP70, has been routinely verified in such studies. In the present study, the effect of NLHS was examined not only for HSP70 but also for *HSC70*, *HSC70-5*, *BIP*, *HYOU1* and *HSPA4*. Resultant data showed that *HSP70*, *HSC70-5* and *BIP* were significantly induced (*P* < 0.05) after NLHS (see Fig. 7). Previous research reported that HSC70-5 is also induced by heat stress in *P*. *vannamei* (and also in response to WSSV virus infection)^15^. As a member of the HSP70 family, HSC70-5/S70P is localised to the mitochondria and confers thermal tolerance by preventing protein aggregation, but also regulates mitochondrial morphology and cellular homeostasis^16^. Similarly, *Lv*HSC70-5 is also localised to the mitochondria and was up-regulated by both heat and cold shock treatments. Therefore, *Lv*HSC70-5 most probably works as a protein chaperone to engage in the tolerance of *L*. *vannamei* to thermal stress. Aquatic invertebrates are mainly dependent on HSP70s, as observed in the genome of *Crassostrea gigas*, which encodes for >88 HSP70s (compared to 17 HSP70s in humans) involved in various cellular protection mechanisms against heat or other stressors^29^. Research in oysters has shown that a HSP70 family member located in the ER can be induced by heat shock^29^. Other research, involving thermal acclimation in *Drosophila melanogaster*, demonstrated the up-regulation of transcriptional activity, and a change in the abundance of HSP70 protein, in flies acclimated to 31 °C (heat acclimation) as compared with flies acclimated at 25 °C. Furthermore, a significantly higher survival rate (*P* < 0.001) was evident in heat-acclimated flies, suggesting that HSP70 plays a more important role in the acquisition of thermotolerance during acclimation^30^. Collectively, existing literature, and our current data suggest that HSP70 family members located in the cytosol (HSP70), mitochondria (HSC70-5) and ER (BIP) are heat shock inducible.

In the TF12 population, obtained after 12 generations of selective breeding for induced thermotolerance, we observed significantly higher expression of *HSP70* and *HSC70* relative to the control population (CF12). This result is related to selective breeding for induced thermotolerance in sunflowers, in which thermo-tolerant lines show enhanced expression of heat shock proteins; the expression of HSP18.1, HSP90 and HSP104 were all induced upon heat-induction^31^. However, the current study reports, for the first time, the expression pattern profile of genes from the HSP70 family during a selective breeding program for induced thermotolerance. Our data demonstrated indicating that these two genes might play important roles in thermotolerance in *Artemia*. Moreover, our results showed that selection can influence the response to NLHS response; selection for induced thermotolerance caused a significant increase (*P* < 0.05) in the expression of *HSP70*, *HSC70* and *HSC70-5* in TF12 after NLHS. On the other hand, HYOU1 and HSPA4 did not show significant up-regulation after NLHS, for both CF12 and TF12 populations. This result might imply that selective breeding for induced thermotolerance could induce the expression of only some HSP70 family members and that these genes were associated with thermotolerance in *Artemia*.

Furthermore, some reports have demonstrated the function of *HSP70*, *HSC70*, and *HSC70-5* in thermotolerance and immune defenses by using RNA interference (RNAi) technique^15,28,32,33^. Iryani *et al*., 2017 used RNAi to verify the role of HSP70 in protecting nauplii of *A*. *franciscana* against abiotic and biotic stressors., The survival of nauplii lacking HSP70, as compared to those containing the HSP70, was decreased 41% by heat stress and 34% upon *Vibrio campbellii* infection^32^ suggesting that HSP70plays an important role in maintaining protein homeostasis by functioning as molecular chaperones while enhancing the host innate immune system against bacterial infection. In insect (*Rhodnius prolixus*), knockdown of HSP70/HSC70 affected insect in starvation or fed conditions and down-regulated the expression of genes related to endoplasmic reticulum stress and immune-related responses before (in starvation) and after the blood feeding^33^. Moreover, in Pacific white shrimp (*P*. *vannamei*), RNAi was used to demonstrate the crucial function of heat shock protein *LvHSP70* in protection against *V*. *parahaemolyticus* AHPND-isolate (VP_AHPND_) after chronic non-lethal heat shock (NLHS). *LvHSP70* knockdown impaired protection against VP_AHPND_ as shown by the increase mortality and higher bacterial counts in the knockdown shrimp compared to the GFP dsRNA injected group. In addition, a significant decrease of hemolymph PO activity in the knockdown shrimp was also observed^28^. Beside HSP70, Yuan *et al*., 2017 also used RNAi to study the functional of *LvHSC70-5* in protection against WSSV infection. The result revealed that shrimp lacking *LvHSC70-5* have increased protein aggregation that enhances the cumulative mortality of WSSV infected^15^. All of these results supported our finding on the possible role of HSP70 family genes, *HSP70*, *HSC70*, and *HSC70-5* in association with thermotolerance and immune responses in *Artemia*.

In summary, this study identified novel genes from the HSP70 family in *Artemia* and highlighted their probable functional association with heat stress. *HSP70*, *HSC70* and *HSC70-5* exhibit responses to heat stress and might represent a potential biomarker for selective breeding for the induced thermotolerance of *A*. *franciscana*. Taken together, these results suggest that those HSP70 family genes possibly contribute to the process of thermal tolerance in *A*. *franciscana*. HSC70, generally accepted to be expressed constitutively, displays increased expression after selection for induced thermotolerance and becomes responsive to a non-lethal heat shock is such population.

## Materials and Methods

### Identification of genes from the HSP70 family in *Artemia*

#### Experimental animals

*Artemia* cysts, originating from San Francisco Bay (SFB, ARC1768), were hatched using an axenic system, beginning with a decapsulation step^34^. Fifty swimming nauplii were transferred to 1-L glass bottles containing 800 mL of sterile 35 g/L artificial seawater (Instant Ocean, VA, U.S.A.) and cultured at 28 °C with constant illumination of approximately 27 μE/m2/s. The animals were fed with live algae (*Tetraselmis suecica*) for 18 days until they reached a juvenile state.

#### Identification of HSP70 family genes using the *Artemia* transcriptome database

In order to identify novel genes from the HSP70 family, we identified phylogenetic reference genes from other species that were as close as possible to *Artemia* and contained the putative conserved HSP70 domain (Table S1). We then used these reference genes in a blast search against an *Artemia* transcriptomic database (unpublished data). Matching nucleotide sequences were used to design specific primers (Table S2). RNA was extracted from juvenile *Artemia* (SFB, ARC1768) using an RNeasy Plus Mini Kit (Qiagen, Hilden, Germany). The purity and quantity of the RNA produced was determined by 2% agarose gel electrophoresis and a NanoDrop 2000 spectrophotometer (Thermo Fisher Scientific, DE, U.S.A.), respectively. First-strand cDNA was synthesised from 1 µg of total RNA using the RevertAid™Hminus First-strand cDNA Synthesis Kit (Thermo Fisher Scientific, DE, U.S.A.). The cDNA was used then for PCR amplification in a total volume of 50 µl containing 1.25 units of DreamTaq DNA Polymerase (Thermo Fisher Scientific, DE, U.S.A.), 1x DreamTaq Buffer, 0.2 mM of each dNTP and 0.5 µM of each primer. After an initial denaturation step at 94 °C for 2 min, the PCR was performed using 30 cycles at 94 °C for 30 s, gene-specific annealing temperatures (Table S2) for 30 s and 72 °C for 3 min; reactions were then terminated at 72 °C for 10 min. The resultant PCR products were purified using a Wizard® SV Gel and PCR Clean-Up System (Promega, WI, U.S.A.), and DNA purity was assessed by agarose gel electrophoresis. DNA concentration was finally measured using a NanoDrop 2000 spectrophotometer before the DNA was sent for sequencing with an automated sequencer (Sanger sequencing) by LGC Genomics (Berlin, Germany).

#### Sequence analysis

The complete ORF sequences of newly identified genes from the HSP70 family were analysed by BLASTX (https://blast.ncbi.nlm.nih.gov) to identify conserved domains and the most similar protein using the following parameters: database, non-redundant protein sequences (nr); organism, all (default); algorithm, blastp (protein-protein BLAST). Next, we performed phylogenetic analysis for each HSP70 family candidate gene. Sequence alignments were performed using ClustalW for the amino acid sequences of the candidate HSP70 family genes based on the deduced amino acid sequences of the presumed orthologous genes in typical species with the highest scores in blastp. ClustalW alignments were performed with two alignment methods: pairwise alignment (10, 0.1) and multiple alignments (10, 0.2); the numbers given in parentheses represent the gap opening penalty and the gap extension penalty, respectively. The delay divergent cut-off was 30%. A phylogenetic tree was then reconstructed using the neighbor-joining (NJ) algorithm and MEGA 7.0 software (https://megasoftware.net). Bootstrap sampling was reiterated 1000 times. Conserved domains were analysed by ExPASy (https://prosite.expasy.org/). Protein molecular mass and theoretical isoelectric point (PI) were also predicted using ExPASy software (https://web.expasy.org/compute_pi/).

### Gene expression analysis in TF12 and CF12 populations

#### TF12 and CF12 populations

In this experiment, we used two *Artemia* populations developed and selectively bred by ARC Laboratory, Ghent University. *A*. *franciscana* cysts, from San Francisco Bay (SFB, ARC1767), were hatched non-axenically. Then, swimming nauplii were collected and divided into two groups. The first group was exposed to a non-lethal heat shock (NLHS) at 37 °C for 30 min and subsequently transferred to 28 °C for 5 h of recovery. These animals were subsequently exposed to a lethal heat shock (LHS) at 41 °C for 10 min. Then, nauplii were transferred back to 28 °C and survivors were collected (on average 1% of the original population and referred to as the TF population). The second group was grown isothermally; this group was referred to as the CF population. Animals from the TF or CF populations were divided equally into five tanks containing approximately 40 L of 35 g/L artificial seawater (Instant Ocean, VA, U.S.A.) with continuous aeration and illumination with approximately 27 μE/m2/s for 16 h per day. Animals were fed with a variety of different feeds, including brown and green microalgae, dried algae, live *T*. *suecica*, and *Artemia* enrichment media. The specific details of the different food sources used are as follows. *Nannochloropsis sp*. (Nanno 3600), *Pavlova sp*. (Pavlova 1800), *Isochrysis sp*. (1800) and *Tetraselmis sp*. were sourced from Reed Mariculture company (CA, U.S.A.). We also used freeze-dried algae, phytobloom freeze dried *Nannochloropsis sp*. (Necton, Olhão, Portugal), enrichment product (Bernaqua, Olen, Belgium) and live algae (*T*. *suecica*, cultured at the ARC Laboratory). We fed the animals on alternate days with a mixture of either *Nannochloropsis sp*. and *Isochrysis sp*. or *Tetraselmis sp*. and *Pavlova sp*. Feeding was carried out automatically several times a day and also during the night. During this time, the diluted algae pastes were maintained at 4 °C and continuous aeration was provided. The animals were always given supplementary feed consisting of dried algae mix and a small amount of live *Tetraselmis*. Dried and live algae were provided manually once a day to each tank. We also provided *Artemia* with an enrichment product. Feeding was always carried out after cleaning. Tanks were cleaned and water was changed daily. The parental generation animals were grown to the adult stage, and the nauplii produced (the F1 generation) were collected and then treated with the procedure given to the parental generation except that the lethal heat shock became more severe every generation (Table S3). Lethal heat shock (LHS) conditions are given in Table S3. Cysts produced by every generation were also collected. The production of C12 and T12 animals took approximately one year (one month per generation, on average).

#### Axenic hatching of TF12 and CF12 populations

TF12 and CF12 cysts were hatched under axenic conditions. After decapsulation step, cysts were then transferred to new 50 mL Falcon™ tubes containing 30 mL of sterile 35 g/L artificial seawater (Instant Ocean, VA, U.S.A.) and cultured in a rotary incubator for 28 h at 28 °C with constant illumination at approximately 27 μE/m2/s. After 28 h, swimming nauplii instar II (mouth is opened to ingest particles) were selected and separated into two groups: one group was used to study gene expression under NLHS treatment. A second group was fed with a variety of feeds, such as brown and green microalgae, dried algae, live *T*. *suecica* and *Artemia* enrichment media and grown for 18 days in 2-L glass bottles containing 1 L and 800 mL of sterile 35 g/L artificial seawater (Instant Ocean, VA, U.S.A.) and cultured at 28 °C with constant illumination of approximately 27 μE/m^2^/s. until they reached the juvenile stage; they were then used for gene expression analysis.

#### Non-lethal heat shock treatment and animal sampling

Swimming nauplii; instar II (approximately 600 animals), from both TF12 and CF12 populations, were divided into two groups, and each group was transferred to a 50 mL glass tube containing 30 mL of sterile 35 g/L artificial seawater (Instant Ocean, VA, U.S.A.) in three biological replicates. Three circulating water baths (Lauda) were set to 60 °C for immediate temperature increase (temperature boost), 37 °C for NLHS and 28 °C for recovery at ambient temperature, respectively. CF12 and TF12 nauplii were exposed to NLHS by immediate incubation at temperature boots (60 °C) to increase the temperature inside the tube; they were then transferred back to the NLHS bath to maintain a temperature of 37 °C for 30 min. Finally, they were moved back to ambient temperature for recovery. CF12 and TF12 nauplii were incubated at 28 °C to act as controls. The treated animals were then passed through an autoclaved filter, and samples were collected after 3 h and 6 h of recovery in each biological replicate tube, and then stored at −80 °C to await gene expression analysis.

#### Gene expression analysis by qRT-PCR

In order to investigate the expression levels of HSP genes in juvenile populations of CT12 and TF12 and the effect of NLHS on selectively bred *Artemia* nauplii and control nauplii, we extracted total RNA from juvenile and nauplii samples using an RNeasy Plus Mini Kit (Qiagen, Hilden, Germany). The purity and quantity of the RNAs produced were determined by 2% agarose gel electrophoresis and a NanoDrop 2000 spectrophotometer (Thermo Scientific), respectively. First-strand cDNA was synthesised from 1 µg of total RNA using the RevertAid™Hminus First-strand cDNA Synthesis Kit (Fermentas).

The qRT-PCR was performed in a StepOnePlus™ Real-Time PCR System (Applied Biosystems) using Maxima SYBR Green/ROX qPCR Master Mix (Thermo Scientific) with specific primers for HSP70 genes: *HSP70*, *HSC70*, *HSC70-5*, *BIP/GRP78*, *HYOU1* and *HSA4* and *Protein disulfide isomerase* (*PDI*), a chaperone activity gene, as a control. The housekeeping gene, *EF1α*, was used as an internal control (Table S2). The cycling parameters started with an initial activation at 95 °C for 5 min followed by 40 cycles of 95 °C for 15 s, 60 °C for 30 s and 72 °C for 30 s. The fluorescent signal intensities were recorded at the end of each cycle. Melting curve analysis was performed from 55 °C to 95 °C with continuous fluorescent reading at 0.5 °C increments to confirm that only a specific product was amplified. Cycle threshold (Ct) values and fold differences for the expression of each gene were recorded by StepOne™ Software (Applied Biosystems). Expression was calculated using the 2^−ΔΔCt^ method, and EF1α as a reference gene^35^. Amplifications were performed in triplicate for each sample. Data were analyzed by two-way analysis of variance (ANOVA) using ‘selection’ and ‘NLHS’ as parameters. This was followed by Duncan’s new multiple range test. All statistical analysis was carried out using SPSS software (IBM, NY, U.S.A.).

## Supplementary information

Dataset 1
