# Supplementary material for: Sequence and expression analysis of HSP70 family genes in Artemia franciscana
Source: Sci Rep. 2019 Jun 10;9:8391. doi: 10.1038/s41598-019-44884-y (PMC6557860; doi:10.1038/s41598-019-44884-y)
Supplement: Supplementary file 1 — Dataset 1 [file 41598_2019_44884_MOESM1_ESM.pdf]

Sequence and expression analysis of HSP70 family genes in *Artemia franciscana*

Wisarut Junprung[1,2], Parisa Norouzitallab[1,3], Stephanie De Vos[1], Anchalee Tassanakajon[2], Nguyen Viet Dung [1,4], Gilbert Van Stappen[1] and Peter Bossier[1,*]

[1]Laboratory of Aquaculture & Artemia Reference Center, Department of Animal Sciences and Aquatic Ecology, Faculty of Bioscience Engineering, Ghent University, Coupure Links 653, 9000 Ghent, Belgium. [2]Center of Excellence for Molecular Biology and Genomics of Shrimp, Department of Biochemistry, Faculty of Science, Chulalongkorn University, Bangkok 10330, Thailand. [3]Laboratory for Immunology and Animal Biotechnology, Department of Animal Sciences and Aquatic Ecology, Faculty of Bioscience Engineering, Ghent University, Coupure 653, Ghent 9000, Belgium.

Current address

[4]Research Institute for Aquaculture No.2, Ho Chi Minh City 71007, Vietnam.

*Corresponding author

Laboratory of Aquaculture & Artemia Reference Center, Department of Animal Sciences and Aquatic Ecology, Faculty of Bioscience Engineering, Ghent University, Belgium. E-mail: Peter.Bossier@UGent.be

Supplementary Table S1: The reference HSP70 family genes from other species

Gene / Species	Accession Number
HSP70 (*Danaus plexippus*)	EHJ73892.1
HSP70 (*Daphnia magna*)	EU514494.1
HSP70 (*Artemia sinica*)	KF683905.1
HSP70 (*Bombyx mori*)	BAF69068.1
HSP70 (*Tigriopus kingsejongensis*)	APH81352.1
HSP70 (*Cotesia chilonis*)	AKA09521.1
HSP70 (*Orius sauteri*)	AIK01869.1
BIP (*Litopenaeus vannamei*)	AFQ62791.1
GRP78 (*Aphis gossypii*)	AKO69637.1
BIP (*Litopenaeus vannamei*)	AFQ62791.1
HSC70 (*Plutella xylostella*)	AFC38439.1
HSC70 (*Metapenaeus ensis*)	ABF20530.1
HSC70 (*Cotesia chilonis*)	AKA09522.1

Supplementary Table S2: Primers used in this study**.**

Primer name	Sequence (5'-3')	Purpose	Annealing (°C)
HSC70-F	GCATTCTTACAATTGAAGAAGGTAT	RT-PCR	55
HSC70-R	ATCGGGGTAGCAAGAACAATCCATTT		
HSC70-5-F	ATGCTGAGTGCATCCAAAGTTTGTT	RT-PCR	55
HSC70-5-R	ACTCCTTCTTCTCTCCTTCTTGT		
BIP/GRP78-F	TTAAACGAAATAAACCAATCAGAATT	RT-PCR	60
BIP/GRP78-R	ATCGGTTGCACTATATCTTCAGCT		
HYOU1-F	ATAGGCTCCTGTCACCGAAACTGAT	RT-PCR	57
HYOU1-R	AGGGAAAATACATGGTCTTGGTTT		
HSPA4-F	AAATCGTTCTACAGCAGCAATAGTTGTCT	RT-PCR	60
HSPA4-R	CCATTATCAAGTCCAGAGGGATCCTGC		
exHSC70-F	ACTTACTCCGACAATCAGCC	qRT-PCR	60
exHSC70-R	CAAATGTCACTTCGATTTGAGGA		
exHSC70-5-F	GAAGGCTCAAGAACAACACC	qRT-PCR	60
exHSC70-5-R	TTCAAATCGTCGACCAATCAAA		
exBIP/GRP78-F	GTGGCTCCACTAGAATTCCTAA	qRT-PCR	60
exBIP/GRP78-R	GTTCACCACTCAAAACTCCAG		
exHYOU1-F	CTAGAAGTTGCTCTTAACAAAGA	qRT-PCR	60
exHYOU1-R	AGATCAGTTAGGTGTGTATAGCAAT		
exHSPA4-F	TGATTTTGGACAATCGGCTTTAC	qRT-PCR	60
exHSPA4-R	CGATATTTGGTCTTAAATTCTCCGA		
exHSP70-F	CGATAAAGGCCGTCTCTCCA	qRT-PCR	60
exHSP70-R	CAGCTTCAGGTAACTTGTCCTTG		
exPDI-F	AGCGTTCATTGAAGATAATGAAGT	qRT-PCR	60
exPDI-R	CATCACTAACGTCATGATCTGC		
EF1α-F	TCGACAAGAGAACCATTGAAAA	qRT-PCR	60
EF1α-R	ACGCTCAGCTTTAAGTTTGTCC		

Supplementary Table S3: The heat shock procedure induction of *A. franciscana* thermotolerance in each generation

Generation	***Heat shock procedure***	
	NLHS	***LHS***
Parental	30 min. 37 °C – 5 h recovery – 20 min. 41 °C	
F1	30 min. 37 °C – 5 h recovery – 10 min. 41 °C	
F2	30 min. 37 °C – 5 h recovery – 13 min. 41 °C	
F3	30 min. 37 °C – 5 h recovery – 15 min. 41 °C	
F4	30 min. 37 °C – 5 h recovery – 18 min. 41 °C	
F5	30 min. 37 °C – 5 h recovery – 20 min. 41 °C	
F6	30 min. 37 °C – 5 h recovery – 20 min. 41 °C	
F7	30 min. 37 °C – 5 h recovery – 20 min. 41 °C	
F8	30 min. 37 °C – 5 h recovery – 25 min. 41 °C	
F9	30 min. 37 °C – 5 h recovery – 30 min. 41.5 °C	
F10	30 min. 37 °C – 5 h recovery – 30 min. 41.5 °C	
F11	30 min. 37 °C – 5 h recovery – 35 min. 41.5 °C	
